# Primary prostate lymphoma: a rare presentation of lower urinary tract symptoms in young aged patient

**DOI:** 10.1093/jscr/rjad598

**Published:** 2024-01-30

**Authors:** Mohammed Ali Alhamadani, Aleksandr Pecherkin, Terrence Doyle

**Affiliations:** Department of Urology, John Hunter Hospital, Lookout Rd, New Lambton Heights, NSW, Australia; Department of Urology, John Hunter Hospital, Lookout Rd, New Lambton Heights, NSW, Australia; Department of Urology, John Hunter Hospital, Lookout Rd, New Lambton Heights, NSW, Australia

**Keywords:** prostate lymphoma, prostate neoplasm, diffuse large B cell lymphoma, non-Hodgkin lymphoma, prostatic specific antigen

## Abstract

Primary prostatic lymphoma is a rare prostate malignancy that accounts for 0.09% of prostate cancer and 0.1% of lymphoma. Clinical misdiagnosis is common as majority of cases present with non-specific urinary symptoms like other prostatic diseases or prostatic cancer. Early diagnosis is of significant paramount as prognosis is dependent on histological type and tumour staging at the time of diagnosis. Urologists should remain aware of rare histological types of prostate cancer as delayed diagnosis of primary prostate lymphoma can be detrimental and lead to advanced disease causing serious sequelae i.e renal failure in addition to the primary pathology.

## Introduction

Lymphoma is a relatively common malignancy which usually originate in the lymph nodes and more rarely in the prostate. Pathologically, lymphomas are divided into Hodgkin’s disease, which is more common, and non-Hodgkin’s lymphoma (NHL), which is a heterogeneous malignancy caused by allogeneic B lymphocytes, T lymphocytes, or natural killer cells [1]. Prostatic lymphoma can be classified as primary or secondary with the former originates from the prostate. Primary prostatic lymphoma (PPL) is a relatively rare malignant tumour affecting the prostate gland, accounting for 0.09% of prostate neoplasms and only 0.1% of newly diagnosed lymphomas [2]. The majority of cases are NHL, whose main pathological type is diffuse large b-cell lymphoma (DLBCL) [1]. Clinical misdiagnosis of prostate lymphoma is common as majority of patients present with non-specific obstructive lower urinary tract symptoms, and early imaging findings are similar to those of prostate cancer [3]. Here in, we report a case of primary prostate lymphoma (DLBCL) in a 25-year-old male who initially presented with undifferentiated lower urinary tract symptoms and progressive worsening of renal function.

## Case report

A 25-year-old male presented with six months of lower urinary tract symptoms including frequency, nocturia, poor urinary flow, dysuria, urgency and occasional urge incontinence. Patient’s comorbidities included iron deficiency anaemia, asthma, ankle and mandibular surgery. He was a non-smoker and occasional ETOH drinker. His only regular medication was pantoprazole for gastroesophageal reflux disease. Physical examination was unremarkable. Digital rectal examination showed diffusely enlarged irregular non tender prostate suspicious for malignancy.

**Figure 1:**
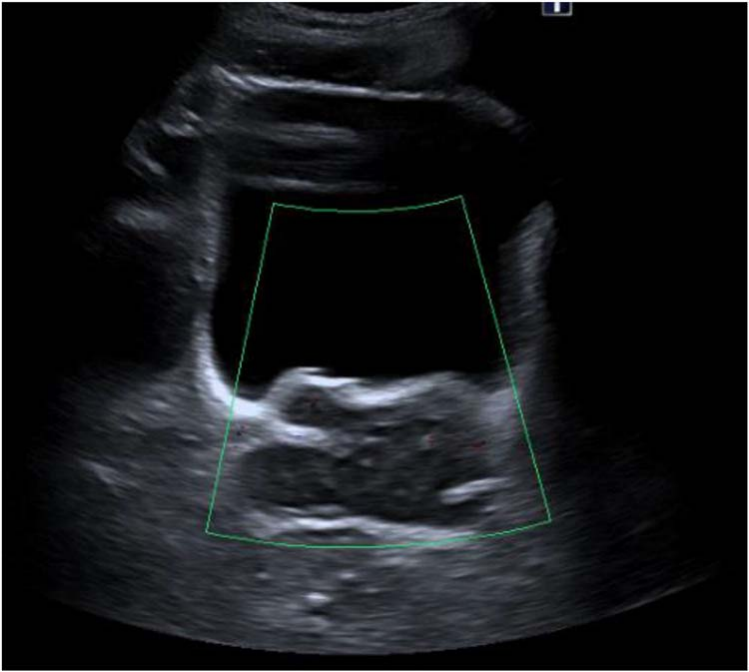
Renal ultrasound showing enlarged prostate indenting into the bladder.

**Figure 2:**
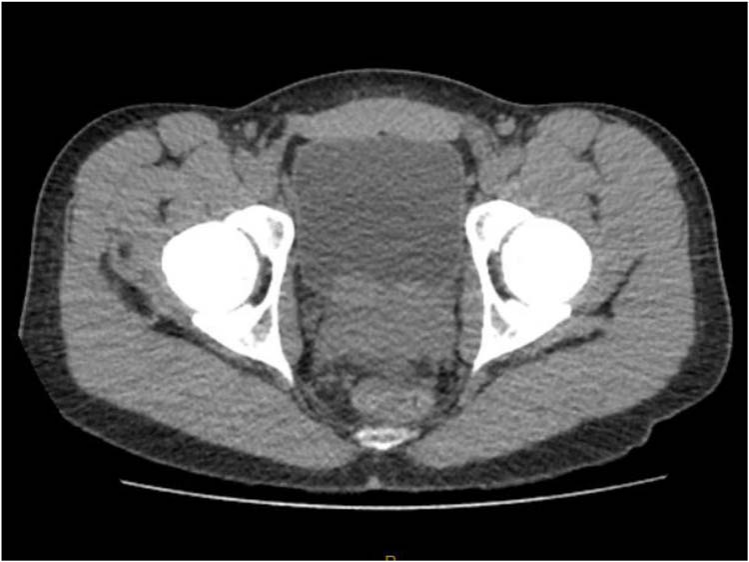
CT KUB showing irregularly enlarged prostate.

**Figure 3:**
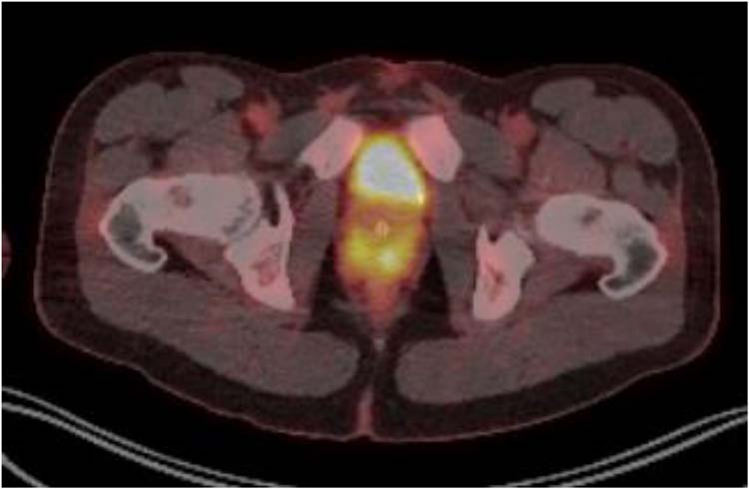
PET FDG scan pre-R-CHOP chemotherapy showing disease activity in the prostate.

Laboratory tests of full blood count and liver function test were unremarkable with prostate specific antigen of 0.53. Mid-stream urine MCS did not show any infection and urine cytology was negative. Renal function test revealed progressive worsening of previously normal eGFR down to 27 ml/min. Initial renal tract ultrasound (Fig. 1) showed enlarged prostate indenting into the bladder base with high post micturition urine residual volume of 185 ml but no hydronephrosis. CT KUB (Fig. 2) showed irregularly enlarged prostate with bilateral hydroureteronephrosis. MRI prostate showed enlarged prostate with PIRADS 5 amorphous mass suspicious for carcinoma involving bilateral seminal vesicles and right vesicouretric bladder. FDG PET (Fig. 3) showed unusual distribution pattern of lymphoma involving the prostate with bilateral symmetrical renal, early pulmonary, and possibly right thyroid lobe involvement.

**Figure 4:**
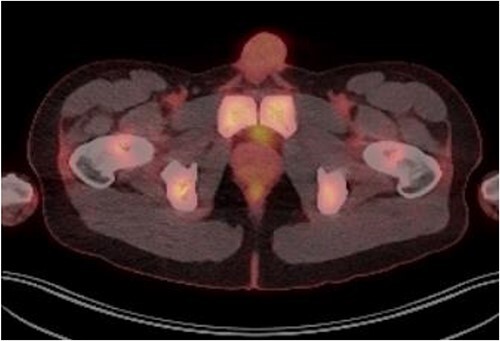
PET FDG scan post R-CHOP chemotherapy showing prostate with complete resolution from the disease.

Patient was catheterised and subsequently underwent bilateral ureteric stenting. Cystoscopic views of the prostate was also abnormal and concerning for malignancy, so transperinal prostate biopsy was performed. Histology of this confirmed diffuse large B cell lymphoma. Renal function improved post ureteric stenting and patient was referred to haematology for management of DLBCL. Progress FDG PET scan (Fig. 4) 3 months post R-CHOP chemotherapy showed complete metabolic response with no avid residual lymphoma.

## Discussion

Prostate lymphoma is classified as primary or secondary according to tumour’s origin with the former originates from prostate [4]. Primary prostate lymphoma is defined using the following criteria: [1] The tumour is confined to the prostate and surrounding soft tissues; [2] there is no lymph node involvement; and [3] no systemic lymphoma is detected at least 1 month after the diagnosis of the primary tumour [5].

Primary prostate lymphoma (PPL) is relatively a rare malignant tumour affecting the prostate gland, accounting for 0.09% of prostate neoplasms and only 0.1% of newly diagnosed lymphomas [2]. PPL is more common in elderly people, with mean age of incidence is 62 years, while secondary prostate lymphoma occurs in relatively younger people [6].

Various subtypes of NHL of the prostate have been reported, including but not limited to follicular lymphomas, Burkitt lymphomas, mantle cell lymphomas, and mucosa-associated lymphoid tissue lymphomas; however, Diffuse large B cell lymphoma remains the most common [7].

Patients with PPL present with non-specific urinary symptoms similar those of other prostatic diseases and hence clinical misdiagnosis is common [8]. Majority of patients present with nonspecific obstructive urinary symptoms such as frequency, nocturia, dysuria, hesitancy, urgency and occasionally as haematuria or acute urinary retention. Specific lymphoma B-symptoms such as fever, night sweats, and weight loss rarely appear in the early stages [3, 5].

The final diagnosis is usually delayed due to the non-specific clinical symptoms at presentation and achieved by pathological and immunohistochemical examinations of samples obtained by transperineal prostate biopsy or transurethral resection of prostate [5]. Cystoscopy, renal and abdominal ultrasound, computed tomography scan, bone scan, bone marrow aspiration, PSA, and LDH are used for accurate staging and to assess the prognosis of the disease. PSA level is usually at lower normal range comparted to prostate cancer elevated PSA [9].

Due to the paucity of reported cases, there is no established consensus for the management. Reported treatment in literatures include radical prostatectomy, radiotherapy, chemotherapy or combination of radiotherapy and chemotherapy [3]. R-CHOP chemotherapy with or without radiotherapy is the treatment of choice. Surgical intervention with radical prostatectomy or TURP does not improve survival rate but it can be of diagnostic value if needle biopsy is equivocal [9]. Most cases reported in recent years were treated with R-CHOP regimen, and in patients with B-cell lymphoma, the addition of rituximab can improve outcomes [2, 3]. Radiotherapy is rarely given alone, but it can quickly relieve symptoms of acute urinary tract obstruction and reduces the rate of local recurrence [3].

The prognosis of prostate lymphoma is poor and is dependent on tumour stage, histologic type, and therapeutic modality. Bostwick and Mann [5] case series of 62 patients with primary prostate lymphoma reported a 1-, 2-, 5-year survival rate of 64%, 50%, and 33% respectively. Tong Fang case series of 29 patients with PPL reported a median overall survival rate of 23 months.

This article reported an extremely rare case of primary prostate lymphoma with unusual presentation of lower urinary tract symptoms and acute renal impairment in a young aged patient. Clinicians and urologists need to keep high index of suspicion for prostate lymphoma when assessing patient with urinary symptoms as early diagnosis is of significant paramount and improve prognostic outcome. More studies will need to be conducted to further evaluate PPL in young aged population [10].

## Data Availability

No data were used to write this case report.

## References

[1] Chen TF , Lin WL, Liu WY, Gu CM. Prostate lymphoma with renal obstruction; reflections on diagnosis and treatment: two case reports. World J Clin Cases 2023;11:1627–33. 36926406 10.12998/wjcc.v11.i7.1627 PMC10011997

[2] Sarris A , Dimopoulos M, Pugh W, Cabanillas F. Primary lymphoma of the prostate: good outcome with doxorubicin-based combination chemotherapy. J Urol 1995;153:1852–4. 7752334 10.1016/s0022-5347(01)67330-0

[3] Ren M , Liu Y. Primary diffuse large B-cell lymphoma of the prostate: a case report and review of the literature. J Med Case Reports 2021;15:546. 10.1186/s13256-021-03143-3 PMC8565014 34727993

[4] Wang K , Wang N, Sun J, Fan Y, Chen L. Primary prostate lymphoma: a case report and literature review. Int J Immunopathol Pharmacol 2019;33:1–4. 10.1177/2058738419863217 PMC6614933 31280618

[5] Bostwick DG , Iczkowski KA, Amin MB, Discigil G, Osborne B. Malignant lymphoma involving the prostate: report of 62 cases. Cancer 1998;83:732–8. 9708938 10.1002/(sici)1097-0142(19980815)83:4<732::aid-cncr15>3.0.co;2-t

[6] Alvarez CA , Rodriguez BI, Perez LA. Primary diffuse large B-cell lymphoma of the prostate in a young patient. Int Braz J Urol 2006;32:64–5. 16519830 10.1590/s1677-55382006000100010

[7] Ezekwudo DE , Ogunleye F, Gbadamosi B, Blankenship LM, Kinoyan M, Krauss D, et al. Primary Extranodal diffuse large B-cell lymphoma of the prostate: a case report. Case Rep Oncol 2017;10:199–204. 28413397 10.1159/000457117 PMC5346921

[8] Hu S , Wang Y, Yang L, Yi L, Nian Y. Primary non-Hodgkin's lymphoma of the prostate with intractable hematuria: a case report and review of the literature. Oncol Lett 2015;9:1187–90. 25663879 10.3892/ol.2014.2829 PMC4315097

[9] Whitmore WF 3rd , Skarin AT, Rosenthal DS. Urological presentations of nonHodgkin's lymphomas. J Urol 1982;128:953–6. 7176059 10.1016/s0022-5347(17)53297-8

[10] Fang T . Clinical analysis of 29 cases with primary malignant lymphoma of the prostate. Chinese Journal of Clinical Oncology 2007;4:129–32.

